# Supplementary material for: mRNA 3′ UTRs direct microRNA degradation to participate in imprinted gene networks and regulate growth
Source: bioRxiv. 2025 Nov 6:2025.11.06.686990. Preprint. [Version 1] doi: 10.1101/2025.11.06.686990 (PMC12637508; doi:10.1101/2025.11.06.686990)
Supplement: 1 [file NIHPP2025.11.06.686990V1-supplement-1.pdf]

bioRxiv preprint doi: https://doi.org/10.1101/2025.11.06.686990; this version posted November 6, 2025. The copyright holder for this preprint (which was not certified by peer review) is the author/funder, who has granted bioRxiv a license to display the preprint in perpetuity. It is made available under aCC-BY 4.0 International license.

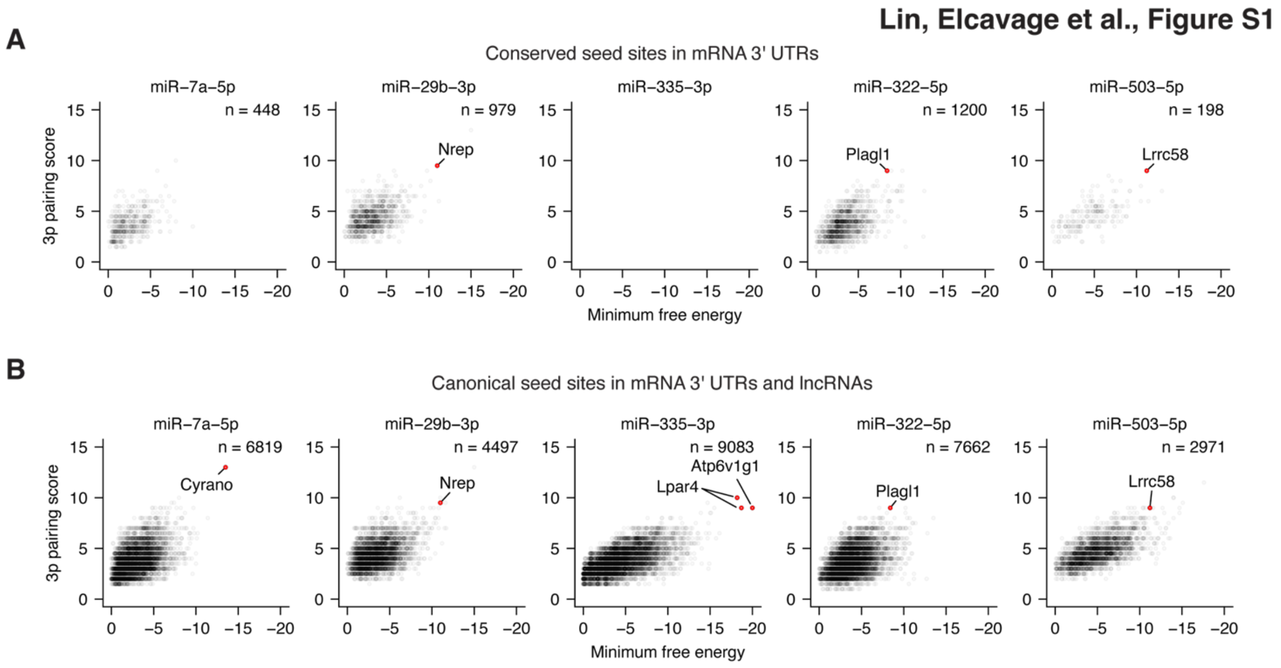

Figure S1. A computational pipeline predicts TDMD trigger sites for miR-335-3p, miR-322-5p, and miR-503-5p. A) Results of a version of the trigger-site prediction pipeline that scores conserved sites. For each conserved seed site (8mer, 7mer-A1, 7mer-m8) in 3′ UTRs, the 3′-pairing score is plotted as a function of predicted 3′-pairing energy. Validated and newly identified TDMD trigger sites are labeled and highlighted in red. miR-335-3p was not analyzed using this pipeline because it had no conserved sites annotated by TargetScan, as it was considered a passenger strand when conserved sites were annotated by TargetScan, and TargetScan does not predict conserved sites of passenger strands. B) Results of a version of the trigger-site prediction pipeline that also scores all non-conserved seed sites in 3′ UTRs and all seed sites in lncRNAs; otherwise, as in A.

bioRxiv preprint doi: https://doi.org/10.1101/2025.11.06.686990; this version posted November 6, 2025. The copyright holder for this preprint (which was not certified by peer review) is the author/funder, who has granted bioRxiv a license to display the preprint in perpetuity. It is made available under aCC-BY 4.0 International license.

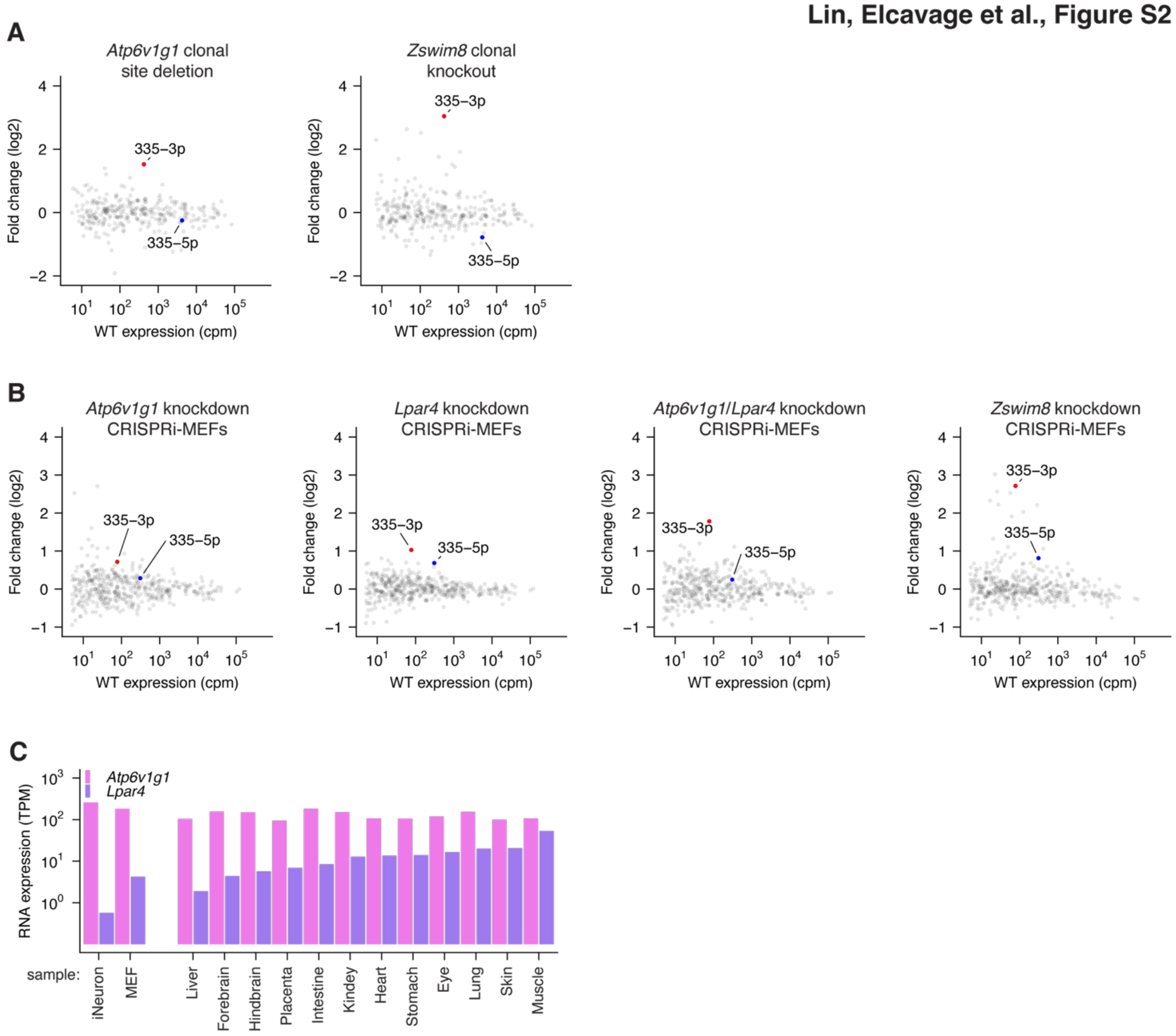

Figure S2. sRNA-seq from cell lines validates *Atp6v1g1* and *Lpar4* as endogenous TDMD triggers. A) sRNA-seq results showing the changes in miRNA levels observed upon disruption of either the miR-335-3p trigger site within *Atp6v1g1* or the *Zswim8* gene. Results from 3–4 clonal lines per genotype were analyzed using DESeq2. The point for miR-335-3p is in red, and the point for the coproduced miR-335-5p strand is in blue. B) sRNA-seq results showing changes in miRNA levels observed when CRISPRi-MEFs were transduced with guides targeting either *Atp6v1g1*, *Lpar4*, both *Atp6v1g1* and *Lpar4*, or *Zswim8*, compared to cells targeted with non-targeting guides. Results from two biological replicates were analyzed using DESeq2; otherwise, as in (A). C) Relative expression of *Atp6v1g1* and *Lpar4* mRNAs, as quantified by RNA-seq in mouse cell lines and embryonic tissues (Shi et al. 2023).

bioRxiv preprint doi: https://doi.org/10.1101/2025.11.06.686990; this version posted November 6, 2025. The copyright holder for this preprint (which was not certified by peer review) is the author/funder, who has granted bioRxiv a license to display the preprint in perpetuity. It is made available under aCC-BY 4.0 International license.

Lin, Elcavage et al., Figure S3

A

```
Atp6v1g1   tctcctttcttacaggtcaggggcgttatctatgaaaaagtagaagtagctattagtaactcctatgaagaagcagccagcctt
–50 nt     tctcctttcttacaggt--------------------------------------------------aagaagcagccagcctt
```

B

```
Lpar4      acagtatttgtgcccaggtcaggagtaaattgaaaaagtaagtgaatagaatagtagcagcaagatatcttaga...
–173 nt    acagtatttgtgccc-----------------------------------------------------------
–69 nt     acagtatttgtgcccaggtcaggagtaaattgaaaaagtaagtgaatagaatagtagcagcaagatatcttaga
–197 nt    acagtatttgtgccc-----------------------------------------------------------

Lpar4   ...gcttatattagtagtttttaaggtggtggttagatagctgtaattttgaaatccatactctcttctgtacattt...
–173 nt    --------------------------------------------------------------------------
–69 nt     gcttatattagtagtttttaaggtggtggttagatagctgtaattttgaaatccatactctcttctgtaca---
–197 nt    --------------------------------------------------------------------------

Lpar4   ...tggagcacattgtagccaaggcgctgctgaatttgtgctcaggtcgggagcatattgaaaaagatgtgtacata
–173 nt    ----------------------------------------aggtcgggagcatattgaaaaagatgtgtacata
–69 nt     ---------------------------------AATTCAGCAGCGGTTGGCTACAATGTGCTCCAAtgtacata
–197 nt    ---------------------------------------------------------------atgtgtacata
```

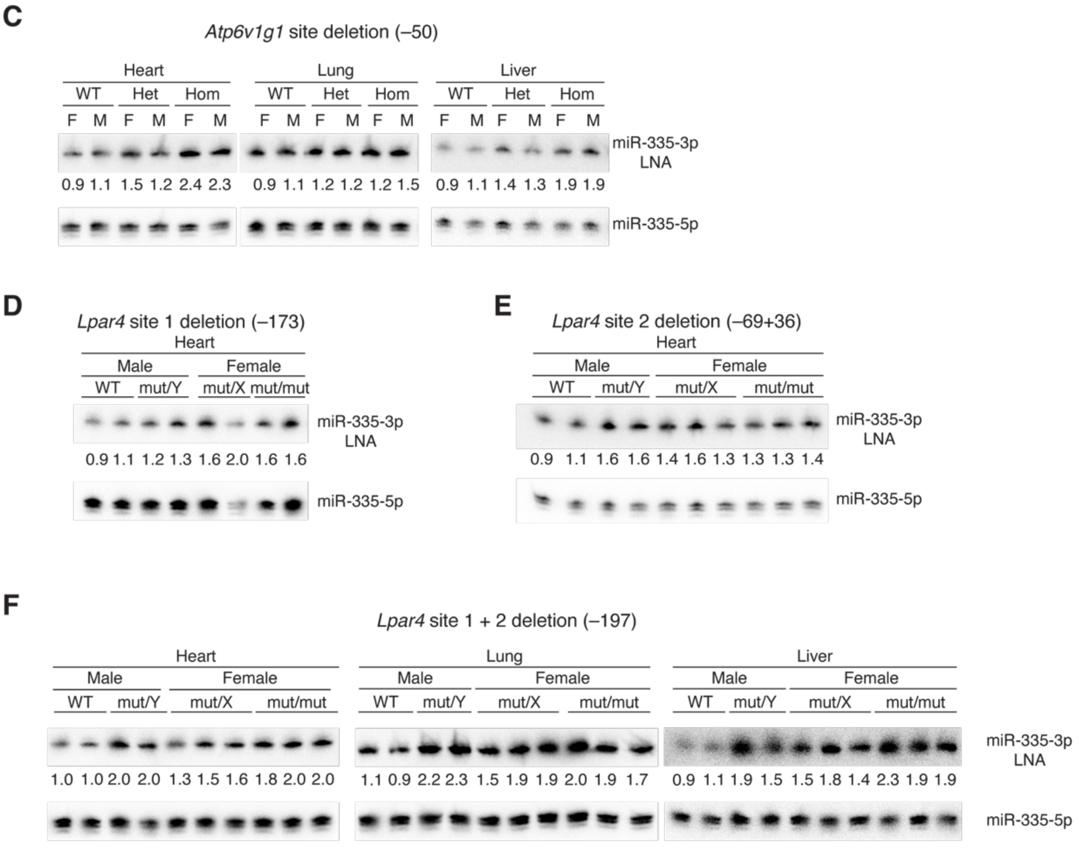

bioRxiv preprint doi: https://doi.org/10.1101/2025.11.06.686990; this version posted November 6, 2025. The copyright holder for this preprint (which was not certified by peer review) is the author/funder, who has granted bioRxiv a license to display the preprint in perpetuity. It is made available under aCC-BY 4.0 International license.

Figure S3. Validation of *Atp6v1g1* and *Lpar4* as TDMD triggers for miR-335 in mice. A) Wild-type and mutant sequences of mouse *Atp6v1g1*. For each gene, the wild-type sequence is shown above the mutant sequence. TDMD sites are in bold; gaps are indicated by dashes; substitutions are in red, and predicted Cas9 cleavage sites are indicated by scissors above the wild type sequences. B) Wild-type and mutant sequences for *Lpar4*; otherwise, as in A. C) Validation of *Atp6v1g1* as a TDMD trigger for miR-355-3p in mice. Shown is a northern blot resolving total RNA of heart, lung, and liver of E18.5 mice, probed for miR-335-3p. Mice were either wild-type (WT), heterozygous, or homozygous for a deletion of the TDMD trigger site in *Atp6v1g1*. Numbers below the lanes indicate the relative fold change, which was obtained by normalizing first to the value of the miR-335-5p loading control and then to the average of the WT samples. D–E) Validation of *Lpar4* as a TDMD trigger for miR-355-3p in mice. Shown are northern blots of RNA from E18.5 heart of *Lpar4* mutant mice in which either TDMD site 1 (*Lpar4*$^{–173}$) or TDMD site 2 (*Lpar4*$^{–69+36}$) has been deleted (panels D and E, respectively); otherwise, as in panel C. F) Effect of deleting both trigger sites within *Lpar4*. Shown are northern blots of RNA from E18.5 heart, lung, and liver for *Lpar4* mutant mice in which both TDMD sites have been deleted (*Lpar4*$^{–197}$). Otherwise, as in panel C.

bioRxiv preprint doi: https://doi.org/10.1101/2025.11.06.686990; this version posted November 6, 2025. The copyright holder for this preprint (which was not certified by peer review) is the author/funder, who has granted bioRxiv a license to display the preprint in perpetuity. It is made available under aCC-BY 4.0 International license.

Lin, Elcavage et al., Figure S4

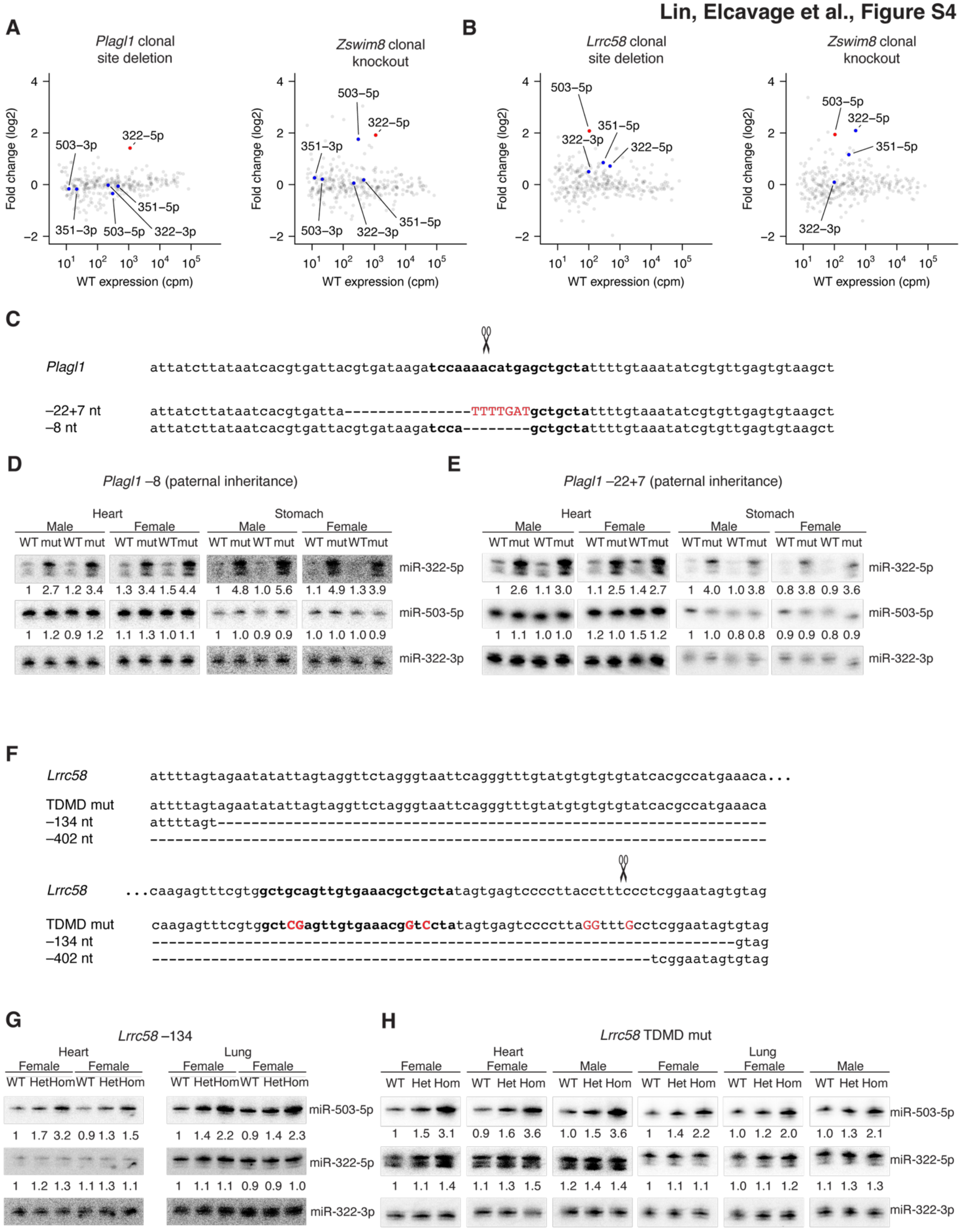

bioRxiv preprint doi: https://doi.org/10.1101/2025.11.06.686990; this version posted November 6, 2025. The copyright holder for this preprint (which was not certified by peer review) is the author/funder, who has granted bioRxiv a license to display the preprint in perpetuity. It is made available under aCC-BY 4.0 International license.

Figure S4. Validation of *Plagl1* and *Lrrc58* as TDMD triggers for miR-322 and miR-503, respectively. A) sRNA-seq results showing the changes in miRNA levels observed upon disruption of either the *Zswim8* gene or the miR-322 TDMD trigger site within *Plagl1*. The point for miR-322-5p is in red, and points for coproduced miR-322-3p, miR-503-5p, miR-503-3p, miR-351-5p, and miR-351-3p are in blue. Results from 3–4 clonal lines per genotype were analyzed using DESeq2. B) sRNA-seq results showing the changes in miRNA levels observed upon disruption of either the *Zswim8* gene or the miR-503 TDMD trigger site in *Lrrc58*. The point for miR-503-5p is in red, and points for coproduced miR-322-5p, miR-322-3p, miR-503-3p, miR-351-5p, and miR-351-3p are in blue. Results from 2–3 clonal lines per genotype were analyzed using DESeq2. C) Wild-type and mutant sequences of mouse *Plagl1*; otherwise, as in Figure S3A. D–E) Validation of *Plagl1* as a TDMD trigger for miR-322-5p in mice. Shown are northern blots of RNA from heart and lung of E18.5 mice with different alleles of trigger-site mutants in *Plagl1* (panels D and E, respectively). Analyses of sex- and litter-matched samples are shown for each of the indicated genotypes. Numbers below the lanes indicate the relative fold change, which was obtained by normalizing first to the value of the loading control (miR-322-3p) and then to the average of the WT samples. F) Wild-type and mutant sequences of mouse *Lrrc58*; otherwise, as in Figure S3A. G–H) Validation of *Lrrc58* as a TDMD trigger for miR-503-5p in mice. Shown are northern blots of RNA from heart and lung of E18.5 mice with different alleles of trigger-site mutants in *Lrrc58* (panels G and H, respectively). Otherwise, as in (D–E).

bioRxiv preprint doi: https://doi.org/10.1101/2025.11.06.686990; this version posted November 6, 2025. The copyright holder for this preprint (which was not certified by peer review) is the author/funder, who has granted bioRxiv a license to display the preprint in perpetuity. It is made available under aCC-BY 4.0 International license.

Lin, Elcavage et al., Figure S5

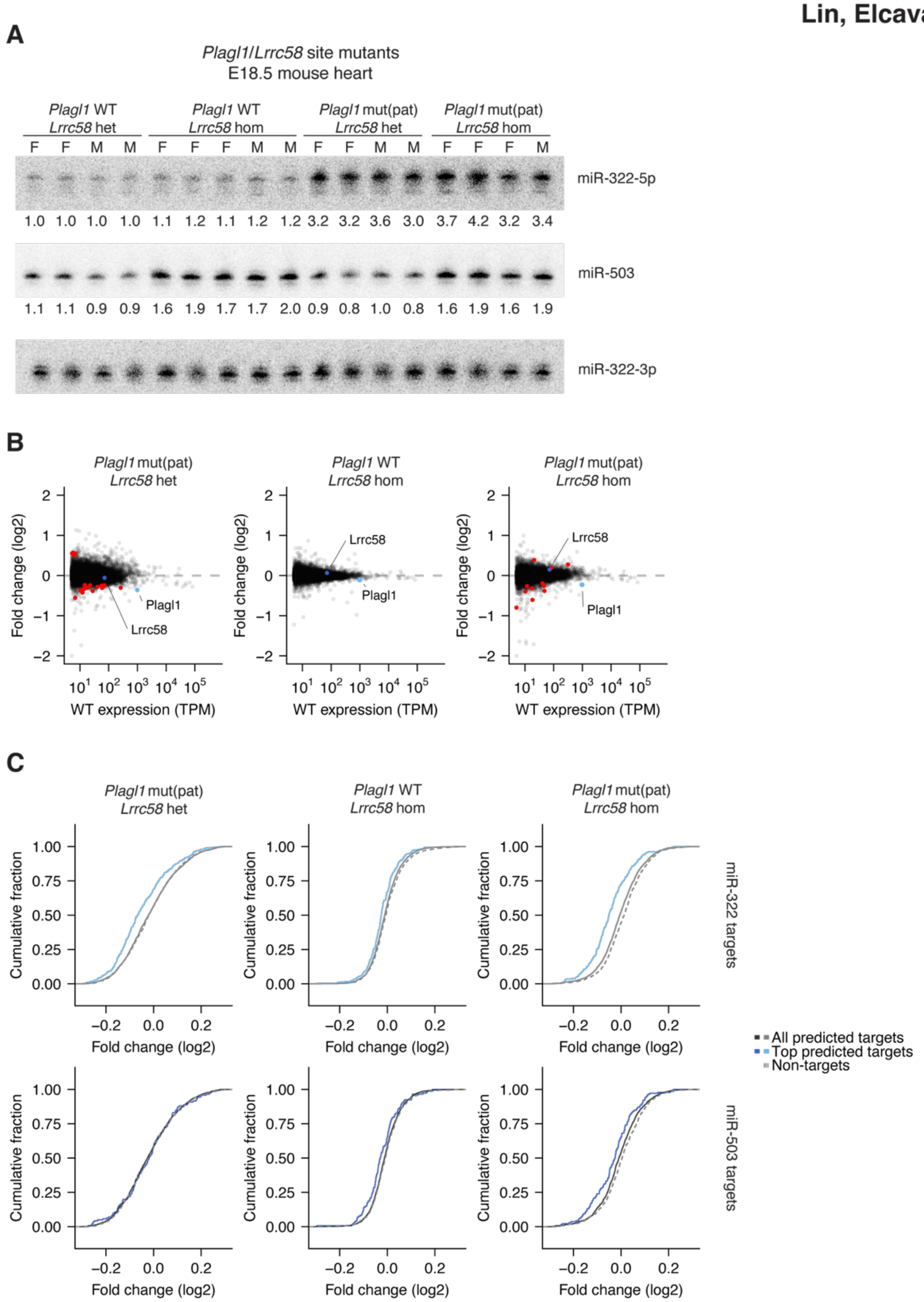

bioRxiv preprint doi: https://doi.org/10.1101/2025.11.06.686990; this version posted November 6, 2025. The copyright holder for this preprint (which was not certified by peer review) is the author/funder, who has granted bioRxiv a license to display the preprint in perpetuity. It is made available under aCC-BY 4.0 International license.

Figure S5. *Plagl1* and *Lrrc58* TDMD trigger sites influence miRNA and mRNA expression in mice. A) Effects of mutating *Plagl1* and *Lrrc58* trigger sites in mice. Shown are northern blots of RNA from heart of E18.5 mice with the indicated trigger site mutants in *Plagl1* and *Lrrc58*. Genotype and sex are indicated above the blots (WT, wild-type; het, heterozygous mutant; hom, homozygous mutant; mut(pat), heterozygous with paternally inherited mutant allele; F, female; M, male). B) RNA-seq results showing changes in mRNA levels observed in mutants of the TDMD trigger sites in *Plagl1* (*Plagl1* mut(pat), *Lrrc58* het), *Lrrc58* (*Plagl1* WT, *Lrrc58* hom), and both *Plagl1* and *Lrrc58* (*Plagl1* mut(pat) *Lrrc58* hom), compared to *Plagl1* WT, *Lrrc58* het littermates. Results from 24 animals (5–7 per genotype) were analyzed using DESeq2. C) The influence of trigger sites within *Plagl1* and *Lrrc58* on levels of miR-503-5p and miR-322-5p predicted targets in vivo. Plotted are cumulative distributions of mRNA fold changes observed in the mutant E18.5 mouse heart relative to wild-type for all predicted targets and top 10% of predicted targets, as determined by TargetScan (Agarwal et al. 2015), and their corresponding control cohorts, matched for 3′ UTR length. For simplicity, only the control cohort corresponding to all predicted targets is displayed (dashed line). The selection of control cohorts was repeated 21 times, and the cohort with the median *P* value (Mann–Whitney U test) is shown here, with this *P* value and the distributions of differences in the median fold changes reported in Figure 2I.

bioRxiv preprint doi: https://doi.org/10.1101/2025.11.06.686990; this version posted November 6, 2025. The copyright holder for this preprint (which was not certified by peer review) is the author/funder, who has granted bioRxiv a license to display the preprint in perpetuity. It is made available under aCC-BY 4.0 International license.

Lin, Elcavage et al., Figure S6

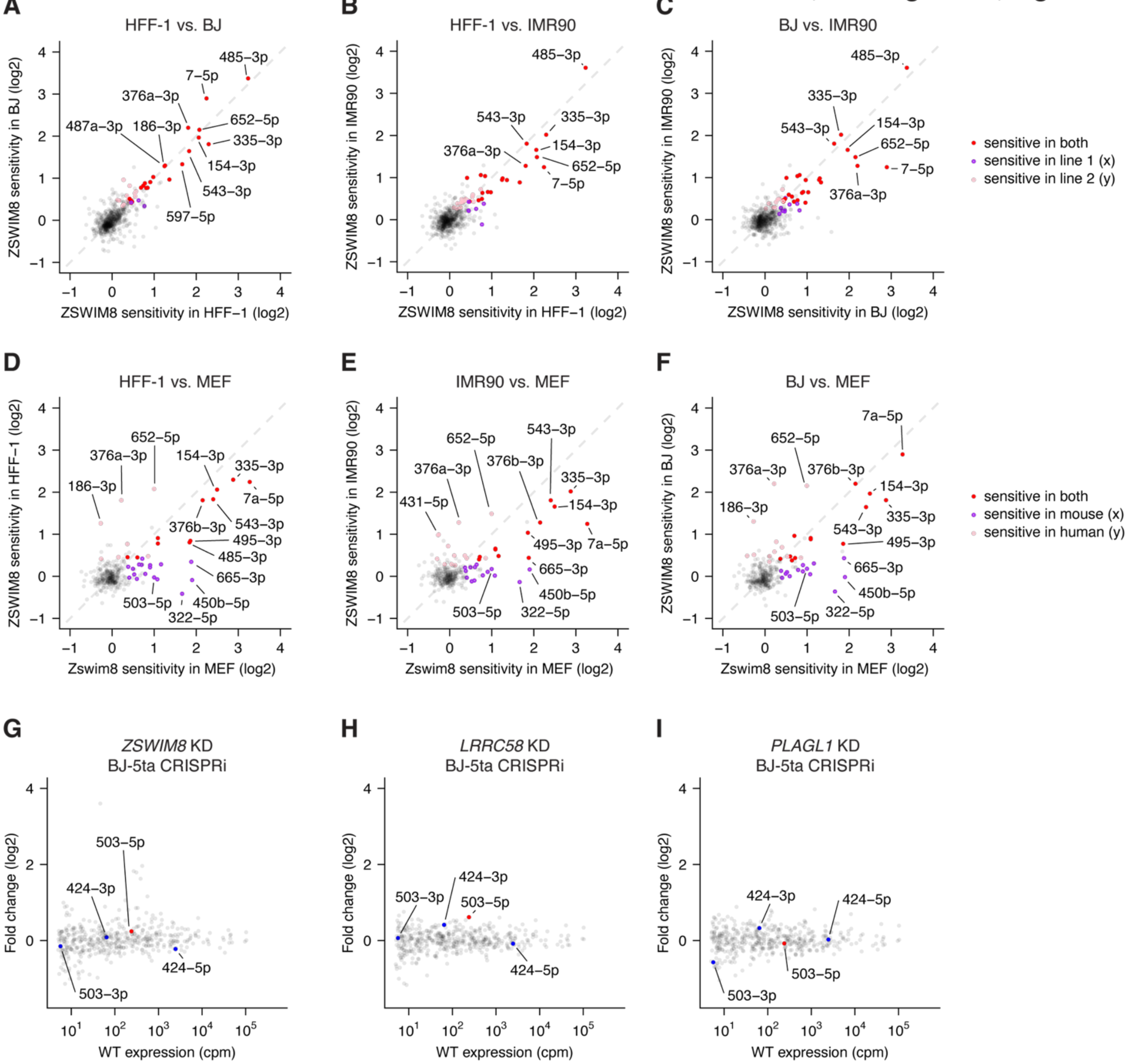

bioRxiv preprint doi: https://doi.org/10.1101/2025.11.06.686990; this version posted November 6, 2025. The copyright holder for this preprint (which was not certified by peer review) is the author/funder, who has granted bioRxiv a license to display the preprint in perpetuity. It is made available under aCC-BY 4.0 International license.

Figure S6. ZSWIM8-sensitve miRNAs are abundant in human fibroblasts. A–C) Abundant ZSWIM8-sensitive miRNAs in three human fibroblast lines. Shown are pairwise comparisons of miRNA fold-changes observed in three different human fibroblast cell lines upon ZSWIM8 knockout, as calculated by DESeq2 (Table S1). Points for miRNAs with statistically significant increases in both cell lines are red (Wang and Bartel 2023). Points for miRNAs with statistically significant increases in only one cell line are purple or pink. D–F) Comparison of ZSWIM8 sensitivities observed between human and mouse. For each miRNA conserved from human to mouse, the fold-change observed in human fibroblast lines upon ZSWIM8 knockout is plotted as a function of the change reported in mouse embryonic fibroblasts (Shi et al. 2023). Colors are as in A. G–H) Conservation of TDMD trigger activity to human cells. Shown are sRNA-seq results plotting miRNA fold changes observed in BJ-5ta CRISPRi human fibroblasts upon knockdown (KD) of either *ZSWIM8* (panel G), *LRRC58* (panel H), or *PLAGL1* (panel I). Results from two biological replicates were analyzed by DESeq2; otherwise, as in Figure S2B.

bioRxiv preprint doi: https://doi.org/10.1101/2025.11.06.686990; this version posted November 6, 2025. The copyright holder for this preprint (which was not certified by peer review) is the author/funder, who has granted bioRxiv a license to display the preprint in perpetuity. It is made available under aCC-BY 4.0 International license.

Lin, Elcavage et al., Figure S7

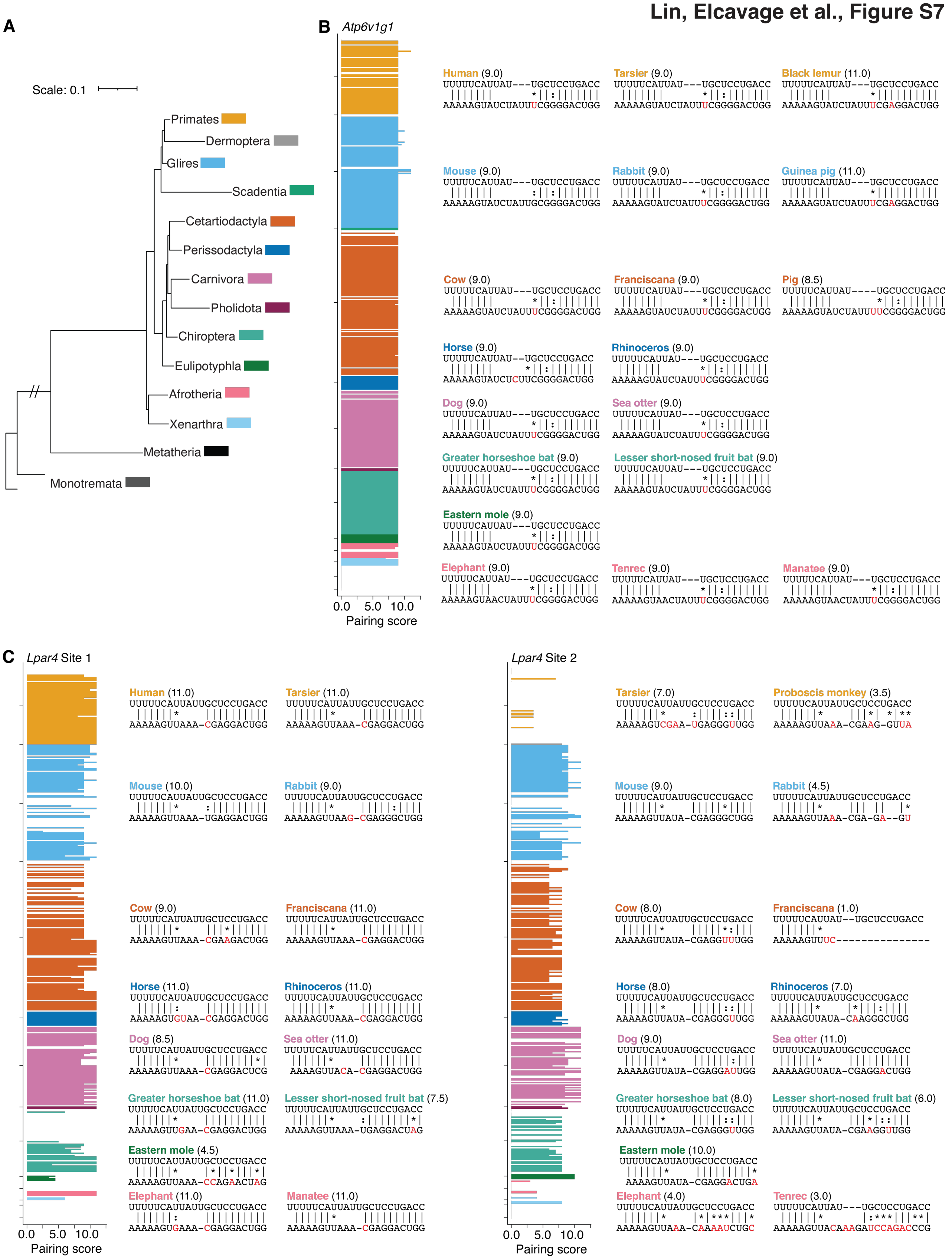

bioRxiv preprint doi: https://doi.org/10.1101/2025.11.06.686990; this version posted November 6, 2025. The copyright holder for this preprint (which was not certified by peer review) is the author/funder, who has granted bioRxiv a license to display the preprint in perpetuity. It is made available under aCC-BY 4.0 International license.

Figure S7. TDMD sites in *Atp6v1g1* and *Lpar4* are evolutionarily conserved. A) Simplified phylogenetic tree of mammalian lineages, based on a 470-species whole-genome alignment (https://hgdownload.soe.ucsc.edu/goldenPath/hg38/multiz470way/) (Letunic and Bork 2024). B) Evolutionary conservation of extensive 3′ complementarity within the miR-335-3p TDMD trigger site in *Atp6v1g1*, across a 470-species mammalian alignment. Each row in the plot corresponds to the 3′ pairing score of our computational pipeline for a species in the 470-way alignment, colored according to clade, as indicated in (A). Missing rows indicate species lacking an orthologous seed match to miR-335-3p. Representative pairing diagrams are shown on the right, with positions that differ from the mouse sequences colored in red. Vertical lines indicate W–C–F pairing; a colon indicates G:U wobble pairing; an asterisk indicates a mismatch. C) As in (B) but for the 2 miR-335-3p trigger sites within *Lpar4*.

bioRxiv preprint doi: https://doi.org/10.1101/2025.11.06.686990; this version posted November 6, 2025. The copyright holder for this preprint (which was not certified by peer review) is the author/funder, who has granted bioRxiv a license to display the preprint in perpetuity. It is made available under aCC-BY 4.0 International license.

Lin, Elcavage et al., Figure S8

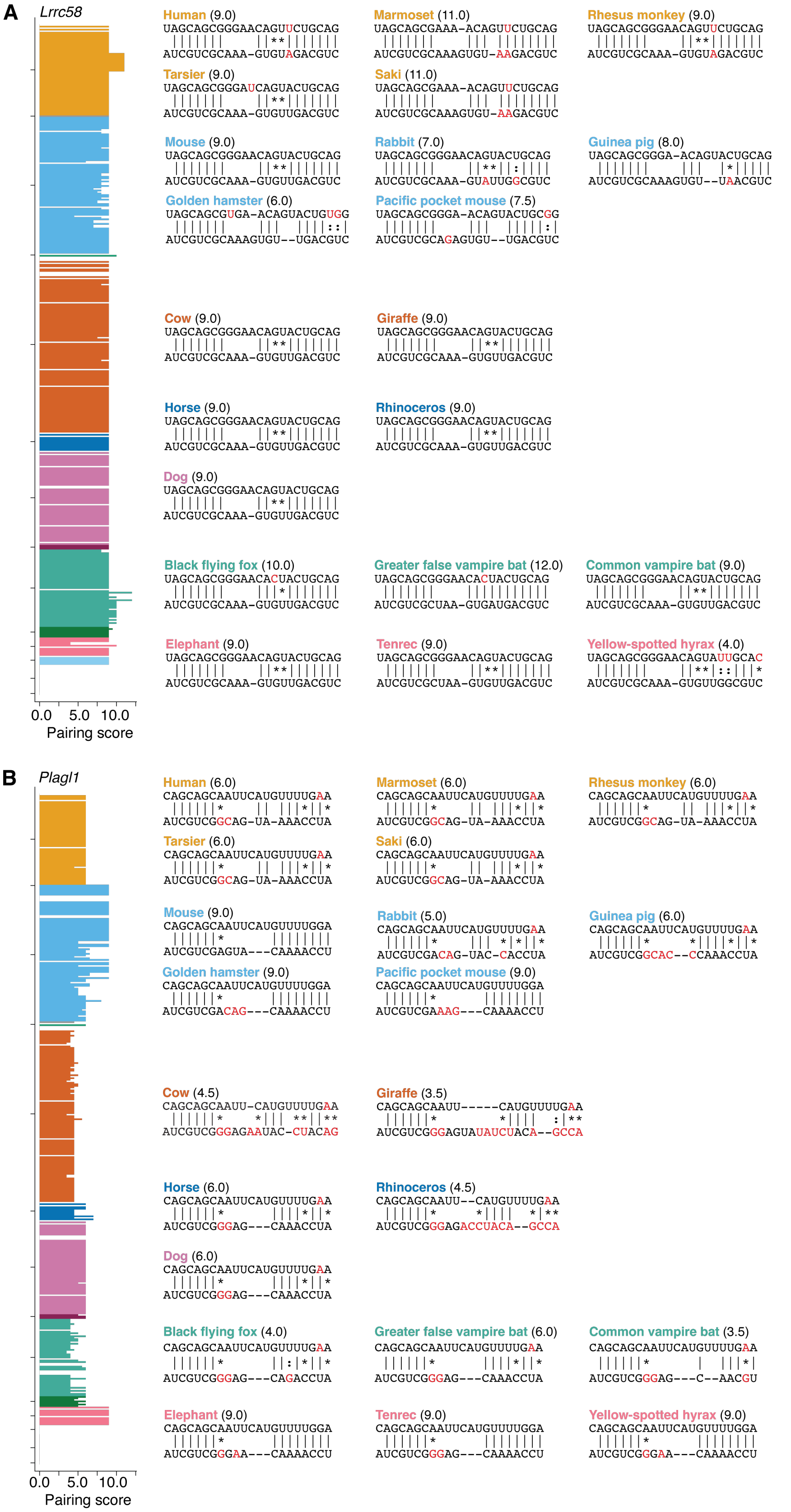

bioRxiv preprint doi: https://doi.org/10.1101/2025.11.06.686990; this version posted November 6, 2025. The copyright holder for this preprint (which was not certified by peer review) is the author/funder, who has granted bioRxiv a license to display the preprint in perpetuity. It is made available under aCC-BY 4.0 International license.

Figure S8. The TDMD sites in *Lrrc58* is more conserved than that in *Plagl1*. A) Evolutionary conservation of extensive 3′ complementarity within the miR-503 TDMD trigger site in *Lrrc58,* across a 470-species mammalian alignment (https://hgdownload.soe.ucsc.edu/goldenPath/hg38/multiz470way/). Missing rows indicate species lacking an orthologous seed match to miR-503. Otherwise, as in Figure S7A. B) As in (A) but for the miR-322/424 site within *Plagl1*.

bioRxiv preprint doi: https://doi.org/10.1101/2025.11.06.686990; this version posted November 6, 2025. The copyright holder for this preprint (which was not certified by peer review) is the author/funder, who has granted bioRxiv a license to display the preprint in perpetuity. It is made available under aCC-BY 4.0 International license.

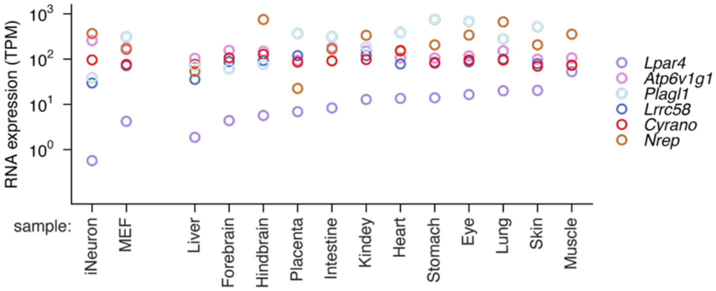

Figure S9. Expression of mammalian TDMD triggers can vary. Relative expression of the six known endogenous mammalian TDMD trigger transcripts, as quantified by RNA-seq in mouse cell lines and embryonic tissues (Shi et al. 2023).
